# Scientific production in sexual and reproductive health and rights research according to gender and affiliation: An analysis of publications from 1972 to 2021

**DOI:** 10.1371/journal.pone.0304659

**Published:** 2024-06-26

**Authors:** Livia Oliveira-Ciabati, Anna Thorson, Vanessa Brizuela

**Affiliations:** 1 Health Innovation Techcenter (HIT), Hospital Israelita Albert Einstein, São Paulo, Brazil; 2 Barão de Mauá University Center, Ribeirão Preto, SP, Brazil; 3 UNDP/UNFPA/UNICEF/WHO/World Bank Special Programme of Research, Development and Research Training in Human Reproduction (HRP), Department of Sexual and Reproductive Health and Research, World Health Organization, Geneva, Switzerland; Emory University School of Medicine, UNITED STATES

## Abstract

**Introduction:**

Peer-reviewed literature is commonly used to assess academic progress and research excellency. However, representation in authorship of global health publications is biased and unfair. In order to shed light on current gaps towards attaining gender equality in scientific production and shift power asymmetries in global health research, we conducted an assessment of authorship trends from 1972 to 2021 with a focus on gender and geographic representation in scientific articles authored or co-authored by researchers affiliated with UNDP/UNFPA/UNICEF/WHO/World Bank Special Programme of Research, Development and Research Training in Human Reproduction (HRP).

**Methods:**

We searched PubMed, Web of Science, and HRP public reports for publications where at least one author was affiliated with HRP. Our main outcome measures were author gender and location of author affiliation, classified by region and country income group. We used descriptive statistics to characterize the publications under analysis as well as the total number of authors from the included papers. We applied a logistic regression model to explore associations between author gender and other characteristics of published articles and a time series analysis to assess how time can influence the inclusion of women as authors in a publication. Python and R were used for all analyses.

**Results:**

A total of 1,484 publications with 14,424 listed authors representing 5,950 unique authors were included in our analysis: 42.5% were female, 35.1% male, and 22.4% unknown (p<0.0001). First authorship was more likely female (56.9%) and from a high-income country (74.6%, p<0.0001) while last authorship was mostly male (53.7%) also from a high-income country (82.5%, p<0.0001). Females more frequently published papers using qualitative data (61.4%) and reviews/estimates (59.4%) while men published more case control (70.7%) and randomised controlled studies (53.0%), p<0.0001. The adjusted odds of there being a female author increased 4% for every additional year that passed.

**Conclusion:**

While there are more females authoring articles as compared to the past, they are still lagging behind with regards to seniority and prestige. Likewise, female representation is closely tied to what institution they are affiliated with and where that institution is located. Global health research institutions need to actively promote change by ensuring women are included in research and research outputs, giving them opportunities to lead.

## Introduction

The UNDP/UNFPA/UNICEF/WHO/World Bank Special Programme of Research, Development and Research Training in Human Reproduction (HRP) hosted at the World Health Organization (WHO) was created in 1972 with a mandate to lead and conduct research and research capacity strengthening activities in sexual and reproductive health and rights (SRHR) [1]. During its initial 50 years of existence, HRP has led in the production of numerous, ground-breaking research in SRHR [2]. The scientific products generated by the programme are sources of guidance in the field of SRHR with an impact worldwide. These include peer-reviewed articles authored by individuals affiliated with HRP in collaboration with researchers worldwide and published in high impact scientific journals [3].

Peer-reviewed publications are extensively used in academia and global health as a way to assess academic progress and research excellency of individuals as well as of the institutions to which individuals are affiliated [4]. However, this measure is not exempt of problems. There is extensive literature on the biased and unfair distribution of authorship in global health publications: papers with no authors from the country where research is being conducted, high-income country (HIC) institutions dominating authorship or authors from low- and middle-income countries (LMIC) being ‘stuck in the middle’ [5–14]. Further, scientific research articles favour English language and global health research focuses on problems as defined by institutions located in HICs [10, 15–17]. Scientific authorship also includes inequities related to pervasive sexism and intersectional effects. While women have consistently taken on leadership roles in global health over the years they continue to lag behind men in academic career progression, as recipients of major research grants, and leaders in research output in major scientific outlets across different country-settings [18–22]. This is reflected in a lower frequency of female authors overall where they are not equally represented in first and last authorship positions [18, 23–26].

Equitable authorship can be framed within the broader discussion on decolonization of global health [27]. While this argument also holds some contradictions and issues (i.e., this is a discussion being held primarily between individuals and institutions based in HICs replicating these power dynamics in their publications and statements) [28], it does present with a framework for asking some difficult questions on research development and global health. Initiatives aimed at social justice and health equity need to acknowledge the persisting patterns of colonialism and intersectional effects including racism and sexism [29, 30]. Despite promising developments with more and more scholars speaking out against these injustices, the current global health field is still very much skewed towards institutions in HICs that define current understanding of health problems, assess their importance and impact, and elaborate potential solutions [27, 31]. While gender equality and universal access to SRHR are included within the Sustainable Development Goals [32] and are a stalwart focus of the World Health Organization, inequalities seen in SRHR research performance by women are telling of the persisting disparities in the global health arena [25, 33].

HRP has been engaged in research capacity strengthening since inception, and since 2017 through the HRP Alliance, promoting issues around gender equality and human rights. HRP is a programme hosted by the World Health Organization, and as such responds to 194 member states and has a key role in convening partners globally and fostering the production of research at the national and sub-national levels. An analysis of scientific production of the special programme HRP could both help shed light on the current gaps in efforts towards attaining gender equality in scientific production and provide the evidence to continue advocating for shifting persisting power asymmetries in global health research. It can also serve as an accountability mechanism of said programme. No prior peer-reviewed publications have focused on authorship trends at the global level, with a focus on papers authored by women and inclusive of country of affiliated institutions in which researchers sitting at the World Health Organization have participated. To address this gap, we conducted an assessment of authorship trends from 1972 to 2021 with a focus on gender and geographic representation in scientific articles authored or co-authored by researchers affiliated with HRP.

## Methods

This is a descriptive-analytical study of publications with at least one author affiliated with HRP, with a specific focus on author gender and affiliation.

### Data sources and screening

We used two separate data sources: journal article databases and publicly available departmental reports. First, we manually searched PubMed and Web of Science using author affiliation as the main search criterion by using the fields relating to “affiliation” or “funding agency” or “text word” to identify the articles. The full search strategy was developed with the help of a WHO Librarian and can be found in **S1 Table**. Second, we manually searched publicly available HRP reports and external evaluations where lists of publications were included; reports were only available from 1986. Our final list included all the articles identified through the PubMed and Web of Science searches, as well as the HRP reports. The full dataset is available in **S1 File**.

We included any article published in a peer-reviewed journal between 1 January 1972 and 31 December 2021 where at least one author was affiliated with HRP. For HRP affiliation, the author could be in any position (including being part of a group/corporate authorship) and the affiliation had to be clearly stated in the paper. We excluded papers that had no authors affiliated with HRP, WHO official publications, WHO guidelines, papers submitted to journals but not accepted, papers in press, book chapters/books, reports, errata and interviews. We further excluded articles for which we were unable to attribute first or last authors’ gender. We acknowledge that this criterium is limited to a dichotomous understanding of gender, which is not reflective of those not identifying with this binary description and does not reflect thinking on this topic by the authors.

To ensure that all articles had at least one author affiliated with HRP, we first extracted author affiliation from PubMed. Second, we performed individual manual checks of all included papers for verification. Third, we developed a rule such that other publications from the same year by the same author(s) were assigned the same affiliation. This was particularly relevant for publications before the early 2010s which is when PubMed began systematically recording author affiliation for all authors and because authors use a variety of ways in which to determine affiliation (e.g., World Health Organization, Department of Reproductive Health and Research, RHR, SRH, HRP, among others). If we were unable to identify at least one author, co-author, or collaborator affiliated with HRP, the paper was excluded from the data analysis.

### Data collection and extraction

**Fig 1:**
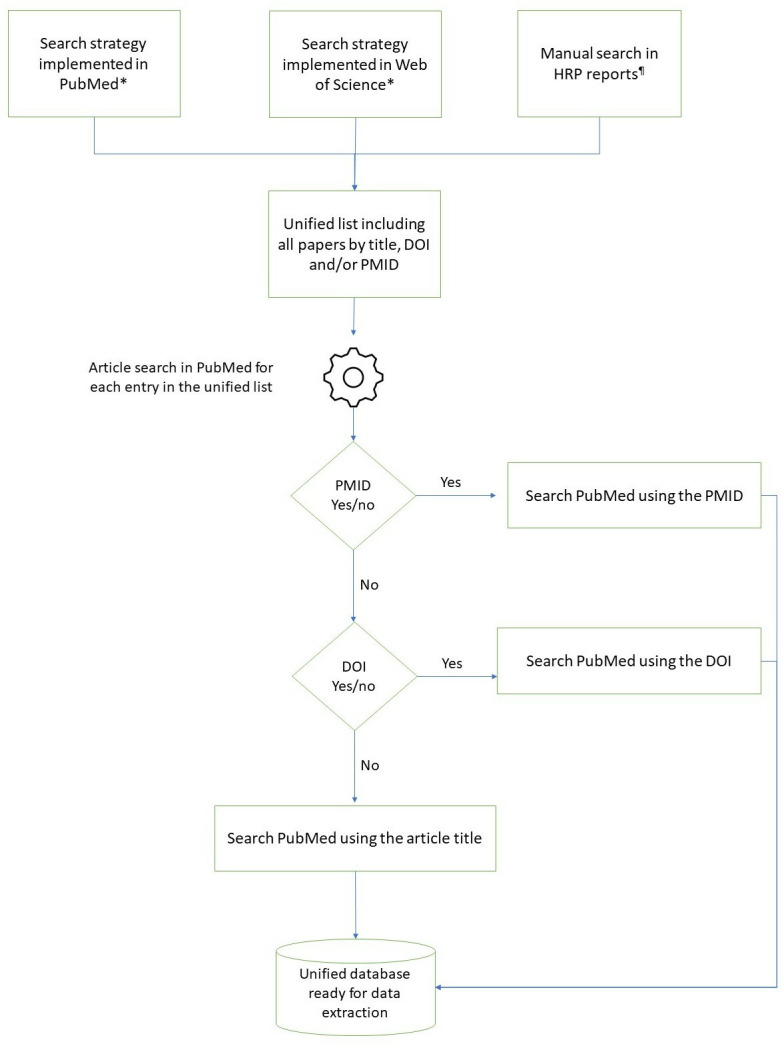
Process for sourcing and identifying publications to be included in this analysis. *For the full search strategy used, see S1 Fig. ¶HRP reports available from 1986 to 2021.

Because we wanted to have similar and comparable information from all articles, we developed an algorithm in Python (Python Software Foundation, Python Language Reference, version 3.1, available at http://www.python.org) based on what others have done for similar purposes [34] to extract data from PubMed. Our algorithm identified all articles from our original list using (i) the unique identifier (PMID) or (ii) the DOI identifier or (iii) the paper’s title (**Fig 1**). In the case of access via PMID, the algorithm generated a url by linking the standard PubMed url and the unique identifier PMID. In the absence of this identifier, the link was generated to perform a search using the DOI or title of the article.

Once we had a unified database of included articles, we extracted the selected variables using the semantic markers of the web page, using the developed Python algorithm. For each article, we extracted 13 variables according to PubMed categories: PMID, title, full list of authors, full citation, journal, publication year, PMCID, DOI, publication type, MeSH terms, and abstract. For each individual author or group author, we extracted the full name, affiliation and authorship position. We ran the algorithm on December 15, 2022.

### Data deduplication and preparation

Once we had the complete list of articles, we removed any duplicated articles based on any of the unique identifiers extracted (PMID, DOI, full citation). We classified the papers according to three categories of authorship: publications attributed to individual authors only, publications on behalf of a group only, and publications with named individual authors on behalf of or for a group.

### Determining gender

For each paper, we extracted the individual names of all authors, including those mentioned as part of a group. From the extracted list of authors, we started a data harmonization process of author names to ensure that all variations were consistent in our database (i.e., names sometimes appearing with only first initial and surname, and at others with spelled out first and middle names as well as names with accents or special characters). To do this data harmonization, we extracted the first letter of the first name and the last name creating a reference standard name. We grouped all the names corresponding to the same standard name and selected the name variation that had (i) highest number of characters and (ii) first name with more than one letter. After, we used this name variation to represent each individual author.

We used similar methods used by others for determining gender [35]. We used the first name of each author to determine gender using a Python library called gender-guesser (https://github.com/lead-ratings/gender-guesser, version 0.3). We decided to use this library as it is quite large, has been used before for other similar analyses, and because it is free of charge, rendering replication of our study more readily feasible by a larger audience. We used gender instead of biological sex because the variable "name" is more closely linked to a person’s perception of gender than to biological sex. Names that did not have their gender automatically identified by the Python library were manually reviewed and checked against other online databases to determine gender (e.g., names.org). Names were classified as: female, male, unknown. This last category included all author names of ambiguity in gender to that most commonly associated with the name or when an author’s name was always indexed with only a first initial or that we were unable to determine manually either. For this reason, and because we could not assign a non-binary category to any of the individuals in this group, we conducted most of the analyses using only entries classified as female or male. We included “unknown” gender to visually inspect the data and conduct sensitivity analyses.

### Determining country of affiliated institution

To determine the country of affiliation, we used the names of institutions where each author was affiliated using a python library called spaCy (https://spacy.io/, version 3.2), which has been proven to be highly accurate for geocoding [36, 37]. We reviewed all cases where it was not possible to automatically assign the country by manually checking for location of institutions using a simple Google search.

### Variable definition

For this analysis, we used the following variables which we describe further in subsequent paragraphs: first and last author gender (for some analyses dichotomized as female/male, for others as female, male, unknown), author position (first, last, co-author, part of a collaborating group), first and last author region and country of affiliated institution, type of publication, year of publication (categorical).

Countries were classified according to country income group using the World Bank (WB) classification for 2022 [38] as low-income, lower-middle income, upper-middle income, and high-income. They were also grouped into WHO regions [39]: Africa, Americas, Eastern Mediterranean, Europe, South-East Asia, and Western Pacific. We classified all HRP affiliated authors into a separate category to avoid skewing the data towards Europe, as HRP is hosted at the World Health Organization headquarters in Switzerland.

For the type of publication, we reclassified PubMed’s categories into three broad categories, according to study design or article category: (i) primary data analyses, (ii) reviews and estimates, and (iii) opinions/methods. Where PubMed classified papers in multiple different categories or these were ambiguous, we manually checked article title and abstract, and where necessary, the methods section to determine an exclusive categorization according to study authors. We included the following publication type papers in the first group: case control study, case reports, cohort study, cross-sectional, intervention/randomised control trial (RCT), mixed-methods study, qualitative study, evaluation, conference paper/proceedings. Reviews and estimates included the following: literature, scoping, and systematic reviews (with or without meta-analysis), estimates, and validation studies. And lastly, we included the following publication types under our category of opinions/methods: comment/correspondence, editorial, statements, study protocols. We assigned “other” to papers that PubMed had no classification for publication type. To ensure accuracy in our reclassification, we checked 10% of the papers with our classification against PubMed’s for consistency.

### Data analysis

Our main outcome measures in this analysis were author female gender and geographical location of author affiliation, classified by region and World Bank (WB) country income group.

For this analysis, we grouped our data in two ways. In one group we had articles as the denominator and in the other group we had authors as the denominator. For the latter, we counted each author every time they appeared in a publication, allowing for multiple inclusions by same individuals. The reason for this was to more closely reflect distribution of gender across individual publications.

First, using the group of data for individual articles, we calculated proportions according to article characteristics based on whether the first author was affiliated with HRP or not. We then calculated proportions regarding type of publication, first and last author gender, and region and country income group according to affiliation. Using the other group of data for individual authors, we calculated author characteristics according to gender, including author position in paper, author affiliation, first and last author region and country income group of affiliated institution, type of publication, and year of publication. We tested for significance between groups using Pearson’s chi-squared test, Fisher’s exact test for count data, and Wilcoxon rank sum test, as applicable.

Second, we applied a logistic regression analysis to explore associations between author female gender (outcome) and other characteristics of published articles (covariates) using the total number of authors across all included publications. We first computed a baseline logistic regression to assess gender by year of publication. We then computed univariate analyses to assess associations between our outcome of interest and the following, individually: year of publication, type of publication, country income group of affiliated institution, author position, and author affiliation. Lastly, we included all the above-mentioned variables in a multivariate logistic regression considering that the odds for being a female author were likely modified by other factors. We calculated crude and adjusted odds ratio with a 95% confidence interval.

Third, to assess how time can influence the inclusion of women as authors in a publication, we conducted time series analyses to assess for trends of gender distribution of authorship from 1972 to 2021. The outcome of interest for these analyses was author gender (female, male, unknown). We first plotted the raw data (number of publications per year, by author gender). We then applied a spline function to smooth data to better represent the trends over time. Using an ARIMA with drift technique, we modelled trend analyses for males and females separately to assess whether publication outputs followed a similar trend by gender, including identifying the size of the trend for each gender. To account for HRP’s staff gender composition potentially skewing our results, we conducted a sensitivity analysis removing all HRP-affiliated authors and plotted the time series without HRP authors from 1972 to 2021.

We used Python programming language and Jupyter IDE (Integrated Development Environment) [40] and R version 4.3.1 (R: A Language and Environment for Statistical Computing, R Core Team, R Foundation for Statistical Computing, Vienna, Austria 2020, https://www.Rproject.org) for statistical analyses and the production of the graphs. The analysis source code for Python is available on GitHub (https://github.com/liviaciabati/Route-A).

This study did not require ethics approval as we used open data that is freely accessible using commonly referenced databases and the study did not involve human subjects.

## Results

**Fig 2:**
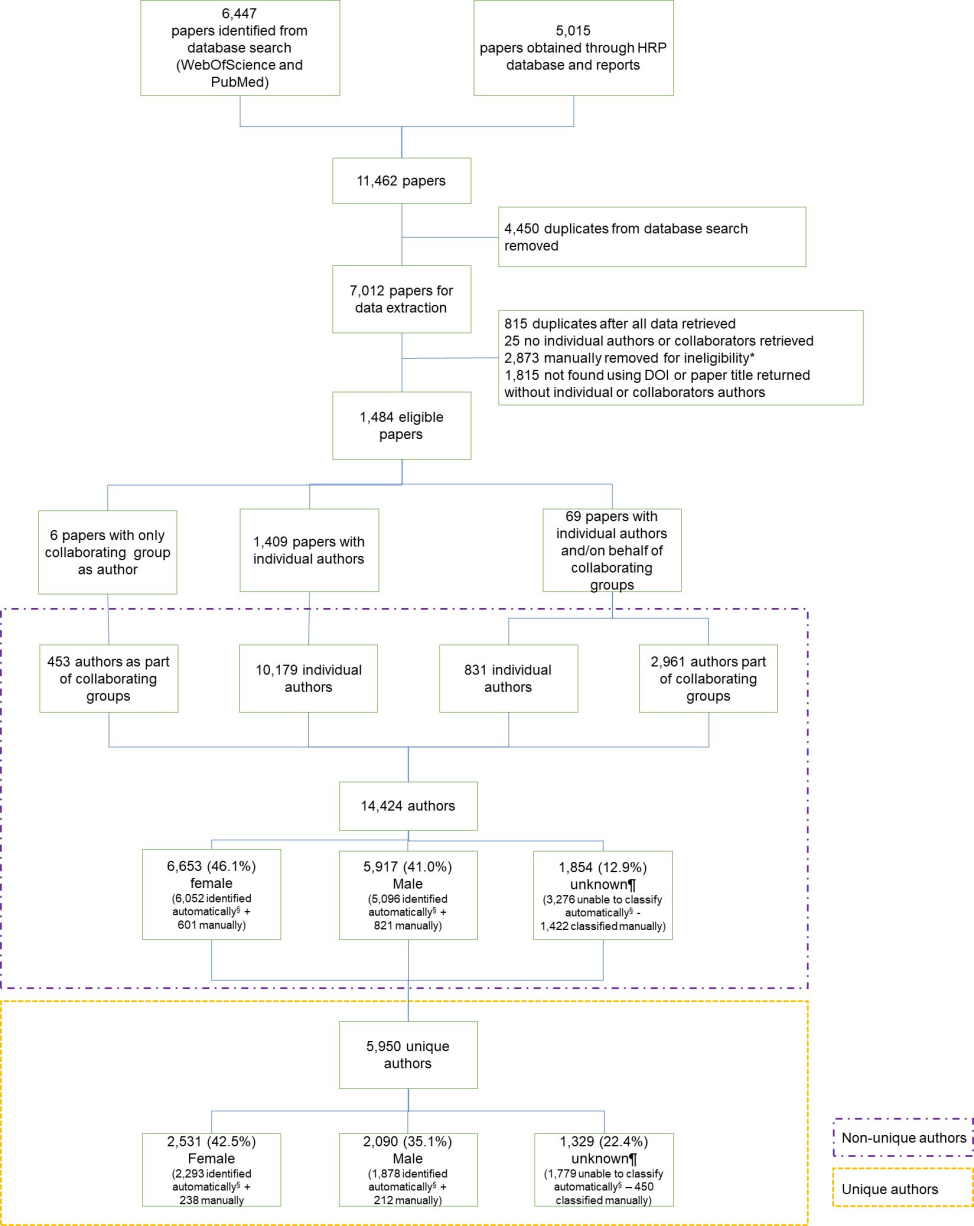
Flowchart of included publications and authors. *Papers where no authors were affiliated with the special programme HRP, WHO publications, WHO guidelines, paper submitted to journals but not accepted, papers in press, book chapters/books, reports, erratum, interviews. ¶Includes all author names we were unable to classify as female or male, § Refers to authors whose gender was identified automatically using the Python gender-guesser library.

Our search resulted in a total of 11,462 articles: 6,447 publications from online database search (PubMed, WebOfScience) and 5,015 from HRP reports (**Fig 2**). We excluded 4,450 duplicates and an additional 5,528 papers based on exclusion criteria: 815 were duplicates identified after all data retrieved; 25 did not have retrievable individual authors or collaborators; 2,873 were manually removed (because no authors were affiliated with HRP, or they were WHO publications, WHO guidelines, papers submitted to journals but not accepted, papers in press, book chapters/books, reports, erratum, interviews); and 1,815 were not found using DOI or paper title or the search returned without individual or collaborator authors. A total of 1,484 publications with 14,424 listed authors (46.1% female, 41.0% male, and 12.9% unknown gender, χ^2^ p<0.001) representing 5,950 unique authors were included in our analysis. The unique authors represented 42.5% women, 35.1% men, and 22.4% of unknown gender (χ^2^ p<0.001). Each author contributed on average with 2.42 papers (range 1–156, SD 5.38). We were unable to assign affiliation to 6,100 (42.3%) authors.

According to individual article’s characteristics first authorship was more likely by a female (56.9%), from an author from the Americas (31.4%) or Europe (26.2%), and an author from a high-income country (74.6%) (**Table 1**). Last authorship, on the other hand was mostly male (53.7%), similarly mostly from Europe (22.0%) or from the Americas (19.9%) and from a high-income country (82.5%). These distributions were similar irrespective of whether first author was affiliated with HRP or not. When the first author was affiliated with HRP, last author was more likely female as compared to articles where first author was not from HRP.

**Table 1:** Characteristics of all included papers, analysed according to first author affiliation with HRP (N = 1,478*).

	Total number of papers		First author affiliated with HRP		First author not affiliated with HRP		p-value^§^
	N	%	N	%	N	%	
**Total number of papers**	**1478**	**100**	**297**	**20.1**	**1181**	**79.9**	
**Type of publication**	1478		297		1181		<0.0001
*Opinions/methods*	*192*	*17*.*0*	*87*	*31*.*3*	*105*	*12*.*3*	
Comment/correspondence	115	59.9	53	60.9	62	59.0	
Editorial	52	27.1	24	27.6	28	26.7	
Statements	6	3.1	2	2.3	4	3.8	
Study protocol	19	9.9	8	9.2	11	10.5	
*Primary data analysis*	*419*	*37*.*0*	*84*	*30*.*2*	*335*	*39*.*3*	
Case control study	11	2.6	1	1.2	10	3.0	
Case reports	5	1.2	3	3.6	2	0.6	
Cohort study	51	12.2	8	9.5	43	12.8	
Cross-sectional	168	40.1	29	34.5	139	41.5	
Intervention/randomized control trial	88	21.0	28	33.3	60	17.9	
Mixed-methods study	20	4.8	4	4.8	16	4.8	
Evaluation study	29	6.9	6	7.1	23	6.9	
Qualitative study	40	9.5	0	0.0	40	11.9	
Conference papers	7	1.7	5	6.0	2	0.6	
*Reviews and estimates*	*520*	*46*.*0*	*107*	*38*.*5*	*413*	*48*.*4*	
Review	453	87.1	98	91.6	355	86.0	
Estimates	62	11.9	8	7.5	54	13.1	
Validation study	5	1.0	1	0.9	4	1.0	
*Other*	*347*	*30*.*7*	*19*	*6*.*8*	*328*	*38*.*5*	
**First author gender** ^‖^	**1307**		**293**	** **	**1014**	** **	0.2969
Female	744	56.9	159	54.3	585	57.7	
Male	563	43.1	134	45.7	429	42.3	
**Region**^**¶**^ **first author**	**1273**		**297**		**976**		<0.0001
HRP**	297	23.3	297	100.0	0	0.0	
Africa	88	6.9	0	0.0	88	9.0	
Americas	400	31.4	0	0.0	400	41.0	
Eastern Mediterranean	18	1.4	0	0.0	18	1.8	
Europe^μ^	333	26.2	0	0.0	333	34.1	
South East Asia	40	3.1	0	0.0	40	4.1	
Western Pacific	97	7.6	0	0.0	97	9.9	
**WB income group**^**‡**^ **of first author country affiliation**	**1273**		**297**		**976**		<0.0001
High income	949	74.6	297	100.00	652	66.8	
Upper middle income	234	18.4	0	0.0	234	24.0	
Lower middle income	75	5.9	0	0.0	75	7.7	
Low income	15	1.2	0	0.0	15	1.5	
**Last author affiliation (n = 900)**	**900**		**179**		**721**		<0.0001
HRP	403	44.8	127	71.0	276	38.3	
Non-HRP	497	55.2	52	29.0	445	61.7	
**Last author gender** ^**‖**^	**1334**		**260**		**1074**		0.0311
Female	618	46.3	136	52.3	482	44.9	
Male	716	53.7	124	47.7	592	55.1	
**Region**^**¶**^ **last author**	**846**		**178**		**668**		<0.0001
HRP**	403	47.6	127	71.4	276	41.3	
Africa	41	4.9	3	1.7	38	5.7	
Americas	168	19.9	23	12.9	145	21.7	
Eastern Mediterranean	6	0.7	1	0.6	5	0.8	
Europe^μ^	186	22.0	20	11.2	166	24.9	
South East Asia	15	1.8	2	1.1	13	2.0	
Western Pacific	27	3.2	2	1.1	25	3.7	
**WB income group**^**‡**^ **of last author country institution (n = 846)**	**846**		**178**		**668**		<0.0001
High income	698	82.5	167	93.8	531	79.5	
Upper middle income	108	12.8	5	2.8	103	15.4	
Lower middle income	31	3.7	5	2.8	26	3.9	
Low income	9	1.1	1	0.6	8	1.2	

*This number includes all papers with individual authors named (n = 1,409) as well as papers with individual authors named on behalf of a collaborating group (n = 69)

^§^Fisher’s exact test for count data or Pearson’s chi-squared test

^‖^For this analysis, gender was dichotomized as female-male, acknowledging that this is reductive and not reflective of people’s gender identity or expression and this dichotomization was conducted using global databases with commonly assigned gender to individual names. Any gender-neutral names or the use of initials or other unknowns were excluded from this analysis.

^¶^Regions organized according to WHO classifications: https://www.who.int/about/structure

**HRP is presented as a region to account for the fact that HRP is physically located at WHO headquarters in Europe

^μ^This excludes all authors affiliated with HRP

^‡^According to the World Bank 2022–2023 country-income classification: https://blogs.worldbank.org/opendata/new-world-bank-country-classifications-income-level-2022-2023

When comparing characteristics of the authors from 1972 to 2021 according to gender, female authors surpassed male authors after 2012, whereas before then, there were more male authors (**Table 2**). Female authors were also more likely from high income country institutions when compared to males (60.5% female vs 39.5% male), a distribution that was opposite among authors from low-income countries (33.0% female vs 67.0% male). 56.9% of first authors were female and 43.1% were male; however, among last authorship this distribution was the opposite. Female first and last authors were mostly from high-income countries (55.0% first, 56.7% last) as compared to males (45.0% first, 43.3% last). Results also depict overall dominance of authors from HIC (69.4%). Publications of articles using primary data was similar between females and males. However, females mostly published qualitative studies (61.4% female vs 38.6% male), case reports (60.9% female vs 39.1% male), and mixed-methods studies (56.2% female vs 43.8% male). Meanwhile, males published more case control studies (70.7% males vs 29.3% females) and intervention/randomised controlled studies (53.0% males vs 47.0% females). Females also published more reviews and estimates than men (χ^2^ p<0.001).

**Table 2:** Characteristics of all named authors and types of publications included in this analysis by gender (N = 12,570*).

	All authors		Female		Male		
	N	%	N	%	N	%	p-value^§^
**Year of publication**	**11480**		**5563**		**5917**		<0.0001
1972–2001	451	3.9	128	28.4	323	71.6	
2002–2011	2102	18.3	1029	49.0	1073	51.0	
>2012	10017	87.3	5496	54.9	4521	45.1	
**Author position on paper**	**12570**		**6653**		**5917**		<0.0001
First	1307	10.4	744	56.9	563	43.1	
Last	1334	10.6	618	46.3	716	53.7	
Co-author^‖^	6902	54.9	3728	54.0	3174	46.0	
Part of a collaborating group	3027	24.1	1563	51.6	1464	48.4	
**Type of publication**	**10215**		**5487**		**4728**		<0.0001
*Opinions/methods*	*1220*	*11*.*9*	*656*	*53*.*8*	*564*	*46*.*2*	
Comment/correspondence	748	61.3	395	52.8	353	47.2	
Editorial	229	18.8	120	52.4	109	47.6	
Statements	41	3.4	22	53.7	19	46.3	
Study protocol	202	16.6	119	58.9	83	41.1	
*Primary data analysis*	*5612*	*54*.*9*	*2822*	*50*.*3*	*2790*	*49*.*7*	
Case control study	41	0.7	12	29.3	29	70.7	
Case reports	23	0.4	14	60.9	9	39.1	
Cohort study	1017	18.1	521	51.2	496	48.8	
Cross-sectional	2605	46.4	1286	49.4	1319	50.6	
Intervention/randomized control trial	1118	19.9	526	47.0	592	53.0	
Mixed-methods study	153	2.7	86	56.2	67	43.8	
Evaluation study	346	6.2	187	54.0	159	46.0	
Qualitative study	290	5.2	178	61.4	112	38.6	
Conference papers	19	0.3	12	63.2	7	36.8	
*Reviews and estimates*	*3383*	*33*.*1*	*2009*	*59*.*4*	*1374*	*40*.*6*	
Review	2756	81.5	1656	60.1	1100	39.9	
Estimates	541	16.0	314	58.0	227	42.0	
Validation study	86	2.5	39	45.3	47	54.7	
*Other*	*2355*	*23*.*1*	*1189*	*50*.*5*	*1166*	*49*.*5*	
**Author affiliated institution**	**12570**		**6653**		**5917**		<0.0001
HRP	1743	13.9	1001	57.4	742	42.6	
Non-HRP	10827	86.1	5652	52.2	5175	47.8	
**Region**^**¶**^ **of author affiliated institution**	**6920**		**3883**		**3037**		<0.0001
HRP^**^	1743	25.2	1001	57.4	742	42.6	
Africa	767	11.1	309	40.3	458	59.7	
Americas	1995	28.8	1174	58.8	821	41.2	
Eastern Mediterranean	101	1.4	46	45.5	55	54.5	
Europe^μ^	1748	25.3	1084	62.0	664	38.0	
South East Asia	221	3.2	97	43.9	124	56.1	
Western Pacific	345	5.0	172	49.9	173	50.1	
**Country income**^**‡**^ **of author affiliated institution**	**6920**		**3883**		**3037**		<0.0001
High income	4804	69.4	2905	60.5	1899	39.5	
Upper middle income	1257	18.2	637	50.7	620	49.3	
Lower middle income	650	9.4	272	41.8	378	58.2	
Low income	209	3.0	69	33.0	140	67.0	
***Region***^***¶***^ ***of*** ***first*** ***author affiliated institution***	**1137**		**668**		**469**		<0.0001
*HRP* ^**^	293	25.9	159	54.3	14	45.7	
*Africa*	67	5.9	24	35.8	43	64.2	
*Americas*	367	32.3	238	64.9	129	35.1	
*Eastern Mediterranean*	18	1.6	8	44.4	10	55.6	
*Europe*	299	26.3	203	67.9	96	32.1	
*South East Asia*	30	2.6	11	36.7	19	63.3	
*Western Pacific*	63	5.5	25	39.7	38	60.3	
***Country income***^***‡***^ ***of*** ***first*** ***author affiliated institution***	**1137**		**668**		**469**		<0.0001
*High income*	879	77.3	549	62.5	330	37.5	
*Upper middle income*	180	15.8	91	50.6	89	49.4	
*Lower middle income*	63	5.6	25	39.7	38	60.3	
*Low income*	15	1.3	3	20.0	12	80.0	
***Region***^***¶***^ ***of*** ***last*** ***author affiliated institution***	**826**		**435**		**391**		<0.0001
*HRP* ^**^	403	48.8	204	50.6	199	49.4	
*Africa*	39	4.7	20	51.3	19	48.7	
*Americas*	164	19.8	92	56.1	72	43.9	
*Eastern Mediterranean*	6	0.7	4	66.7	2	33.3	
*Europe*	179	21.7	101	56.4	78	43.6	
*South East Asia*	13	1.6	7	53.8	6	46.2	
*Western Pacific*	22	2.7	7	31.8	15	68.2	
***Country income***^***‡***^ ***of*** ***last*** ***author affiliated institution***	**826**		**435**		**391**		0.0242
*High income*	685	82.9	368	53.7	317	46.3	
*Upper middle income*	105	12.7	51	48.6	54	51.4	
*Lower middle income*	29	3.5	16	55.2	13	44.8	
*Low income*	7	0.9	0	0	7	100.0	

*Includes only female (n = 6,653) and male (n = 5,917) authors

^§^Pearson’s chi-squared test or Wilcoxon rank sum test

^‖^Co-author means an author in any position other than first or last

^¶^Regions organized according to WHO classifications: https://www.who.int/about/structure

**HRP is presented as a region to account for the fact that HRP is physically located at WHO headquarters in Europe

^μ^This excludes all authors affiliated with HRP

^‡^According to the World Bank 2022–2023 country-income classification: https://blogs.worldbank.org/opendata/new-world-bank-country-classifications-income-level-2022-2023

**Fig 3:**
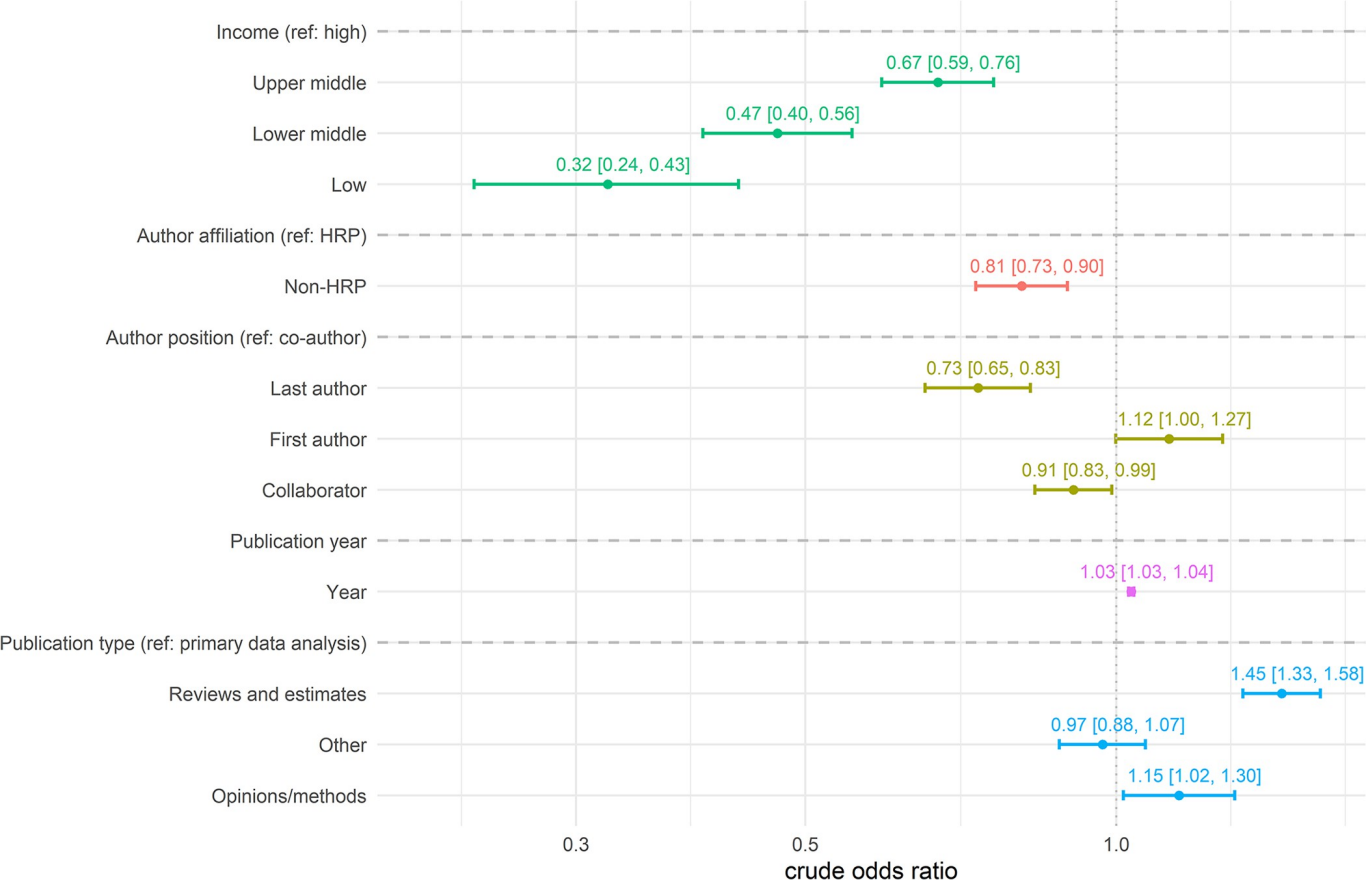
Forest plot indicating the odds of having a female author given certain characteristics of the authors and the papers they published (N = 12,570).

Our univariate logistic analysis using the entire population of female and male authors shows that most covariates were significantly associated with author gender (**Fig 3**). The odds of having a female author overall participating in an article published in a peer-reviewed journal increased 3% for every additional year between 1972 and 2021 (cOR 1.03, 95% CI 1.03–1.04). Similarly, the odds of having a female author were significantly higher for reviews and estimates, as compared to publications using primary data (cOR 1.45, 95% CI 1.33–1.58). However, the odds of there being a female author overall among all published papers decreased by 68% if the author was affiliated with a low-income country, 53% with a lower middle-income country, and 33% with an upper middle-income country as compared to a high-income country. When compared to being a co-author not in first or last position, the odds of having a female author were lower for senior authorship (cOR 0.73, 95% CI 0.65–0.83). And lastly, not being affiliated with HRP fared worse for women authors (cOR 0.81, 95% CI 0.73–0.90).

After assessing for effect modification of author gender by year of publication, type of publication, country income group, author position, and author affiliation, we found that the odds of being a female author followed the same trend as in the univariate analyses (**Table 3**). The odds of having a female author in any position increased 1.04 times for every additional year (95% CI 1.03–1.06) and were still lower when considering last authorship position (aOR 0.79, 95% CI 0.67–0.92) as compared to being a co-author. The odds of being a female author was significantly lower for those affiliated with an institution in a low- (aOR 0.32, 95% CI 0.24–0.43), lower-middle (aOR 0.45, 95% CI 0.37–0.52), or upper middle-income country (aOR 0.66, 95% CI 0.58–0.76) as compared to a high-income country. Female authors were 41% more likely to be included in publications falling under the reviews and estimates category (aOR 1.41, 95% CI 1.25–1.60) as compared to papers using primary data.

**Table 3:** Association between characteristics of the authors and publications, and female authorship.

*Variable*	*aOR*	*95% CI*	*p-value*
Year of publication	1.04	1.03–1.06	<0.001
Country income group			
High income	REF	-	-
Upper middle income	0.66	0.58–0.76	<0.001
Lower middle income	0.45	0.38–0.54	<0.001
Low income	0.32	0.24–0.43	<0.001
Author position			
Co-author	REF	-	-
First	1.18	1.02–1.37	0.025
Last	0.79	0.67–0.92	0.002
Part of a collaborating group	0.89	0.73–1.08	0.2
Author affiliation			
HRP	REF	-	-
Non-HRP	1.12	0.99–1.27	0.080
Type of publication			
Primary data analysis	REF		
Opinion/methods	1.08	0.92–1.26	0.4
Reviews and estimates	1.41	1.25–1.60	<0.001
Other	1.09	0.94–1.27	0.2

aOR: adjusted odds ratio

Adjusted for year of publication (categorical variable), country income group (categorical variable), author position (categorical variable), author affiliation (dichotomous variable), type of publication (categorical variable)

**Fig 4:**
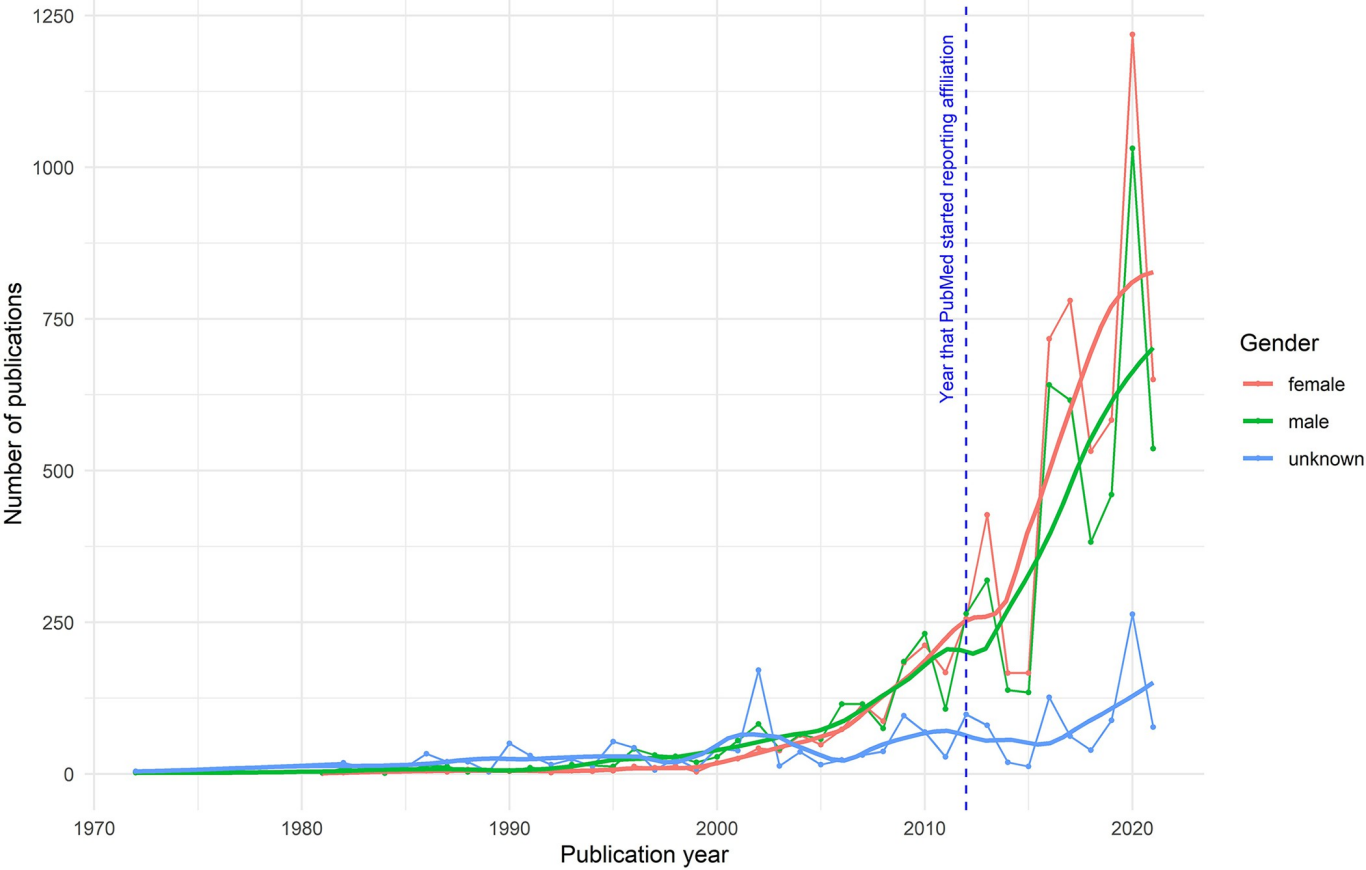
Time series analysis of all authors included in publications from 1972 to 2021, representing absolute and smoothed data, stratified by gender.

Our analysis of publications over time according to gender tell a similar story. The plotting of all authors contributing to publications from 1972 to 2021 over time show that the number of female authors increased, slightly surpassing men in later years (**Fig 4**). When analysing the trends over time of authors included in publications stratified by gender, we found a similar increasing and consistent trend. However, the difference for females is larger than for men (drift coefficient: 21.15 for females vs. 15.27 for males–results not shown). For females and males there was a marked increase in data in 2012, which corresponds to when PubMed began systematically reporting on all author’s affiliations. Results from our sensitivity analysis showed a very similar picture (S1 **Fig**). Even after removing all HRP-affiliated authors, the difference for females continued to be larger than for men, yet these numbers were lower and the difference between them was also reduced (drift coefficient: 17.19 for females vs. 13.51 for males–results not shown).

## Discussion

This paper evaluated 50 years of articles published in peer-reviewed journals by researchers affiliated with HRP according to gender, authorship position, and authors’ country of affiliation. We found that while publication output increased over time for males and females, the presence of female authors in peer-reviewed papers significantly increased with every year that passed between 1972 and 2021. Female authors were less likely senior authors across all country income groups, a difference that was even more pronounced among authors from low-income countries. However, being affiliated with HRP was favourable for female authors.

Research output as expressed through peer-reviewed publications has been used for decades in many settings -including in academia and global health- as a way to assess research excellency in individuals and at group/institutional level [9]. Our analysis showed a positive outlook on female authorship, a trend that seems to be growing over the years, in line with what others have found in the past [41–44]. Even if our analysis may have missed publications prior to 2012 when PubMed began systematically recording affiliation of all authors, in general, publication outputs have increased over time and across the board, supporting our finding of increased publications. Moreover, our time series analysis seems to show that this positive trend has been favouring women over recent years, which supports what others have found [41–43, 45].

However, senior authorship, a metric that is important for academic progression, continues to be held primarily by men, perhaps signalling prevailing gender norms whereby men continue to sustain positions of power and decision-making [21, 35, 46, 47]. Importantly, papers where first author was affiliated with HRP were more likely to also have a female senior author. Further, we found more female first authors across all papers published between 1972 and 2021, while others found the opposite [43]. In fact, other analyses of gender and authorship found a heightened inequality resulting from the recent COVID-19 pandemic which disproportionately affected women [48–50]. While our analysis included years of pandemic, we did not see any dip in overall presence of female authors across publications during these years, despite what others have found [49]. We are aware, nonetheless, that publications of projects initiated during the pandemic may not have been included in our analysis given our cutoff date.

Our findings show that women were less likely to author papers on case control or intervention/randomised controlled studies (RCT), designs that sit at the top of the “evidence hierarchy” [51]. Moreover, qualitative research is oftentimes and anecdotally seen as “less rigorous” or “less prestigious” than their quantitative counterparts. While we disagree with this assumption, and recent WHO guidelines have highlighted the critical importance of listening to people’s voices through qualitative research, RCTs are still viewed as more significant in many medical and public health research circles. Our analysis slightly favoured females regarding authorship of commentaries and editorials, yet evidence from others shows that men author more editorials or are more often invited to write comments [24, 52]. Similarly, others have found that men more often sit at editorial boards of scientific journals and women continue to lag behind in senior academic positions [53–55]. Interestingly, another study found that women were more likely than men to have open and frank discussions on authorship, authorship position, and inclusion in different parts of the research process [47]. This may help also explain why last authorship positions, those indicating more seniority and prestige, continue to be held by men.

Country of affiliation (relating to country income group and region) appeared to be another important factor in determining the presence of female authors in a scientific publication on SRHR. Importantly, we found that being affiliated with HRP was particularly significant. This was perhaps expected given the tenets of gender equality and human rights upon which the programme is built [56], together with the focus on SRHR which often requires a particular lens on gender and the fact that the department has a large proportion of female researchers. Aside from time, *where* the institution a woman was affiliated with was located (i.e., high-income country vs low-income country, HRP vs not-HRP) impacted whether she would be included as an author, especially in first and last authorship positions. Others have also found that while women’s presence as first author increased over the years, this was mostly seen in North America and Europe [41, 57]. In fact, our analysis also showed that high income countries had the most female authors, something that continues to reflect the persisting inequality in access to academic publishing for women across the world. Women researchers from low-income and lower-middle income countries have fewer chances at both being included as an author but even more so of being in a senior leadership position [35]. Evidence shows that high-income countries concentrate the vast majority of the authors of research production [57], and especially when considering last authorship [13, 14]. Intersecting gender and geographic location, the discrepancy becomes more evident as the country’s income level decreases, with few women authors from low-income countries. While time seems to be a good indicator of increased presence of female authors, unless the global health community stands up for women globally -especially those affiliated with institutions in low- and middle-income countries- the disparities will likely continue.

### Strengths and limitations

Our analysis contributes to the growing literature examining authorship across journal publications. Our focus on publications from a leading global health institution with a specific mandate on sexual and reproductive health and rights research and research capacity strengthening offers a unique opportunity to assess the evolution of the inclusion of women in research leadership positions over time. We included thousands of entries in our analysis and used systematic, automated data collection methods thus reducing errors in the transfer of information. We also conducted several data quality checks, using automated and manual methods to ensure minimal errors. However, our analysis has some limitations. First, while PubMed is one of the main sources of access to scientific literature, it proved limited. The non-systematic presence of affiliation data for all authors prior to 2012 including for many co-authors and individuals that were part of group authorship, and the great variability of keywords and different names for the same author were some of the difficulties encountered. This may have led to a loss of relevant information and an under-representation of certain authors or institutions, especially in those years from 1972 to 2012. Second, we determined gender by means of individual’s given name. While we used a large library source that includes names from all over the world, it includes fewer entries for names from smaller regions, countries or communities (or those considered minorities), as well as from Asian countries, and has limited access to names from places where censuses are not carried out. This likely resulted in bias favouring names from high income, Western countries. However, our rigorous use of manual checks for shortcomings of the programmes used gives us confidence in our findings. Further, gender is not always related to the name, as gender identity is complex and multifaceted and given that gender is not a binary concept, the authors are aware of how limited this concept is employed in this study. Unfortunately, until we start systematically collecting data on gender both in global health research and in publishing, a more accurate analysis of gender will remain a challenge. And third, our classification of article type was based on descriptors provided by PubMed and checked manually according to what was described in the paper title or abstract or methods. This means that some articles may have been misclassified. Relatedly, while other researchers may have chosen to classify papers in other ways, ours was one such way to do so. However, using PubMed’s database as a starting point is a strong suit as this is a very large database that people consult frequently and are thus accustomed to its classification systems. And lastly, given the focus of our analysis on publications where at least one co-author was affiliated with HRP whereby each unique author was named in over two publications, we may be overstating the average contribution of individual authors to SRHR research.

### Implications for future research

We emphasize the need to constantly measure the gender and geographic gap in scientific production and suggest that all research institutions conduct analyses like this as a mechanism for accountability and monitoring of progress. In pursuit of gender, ethnic, religious and cultural equality, a data-driven approach is needed, constantly measuring, setting targets and creating plans to accelerate the progress. For example, funders should request that researchers located in the settings where studies are being conducted take on leadership roles, including considerations for gender equality [58]. Further, journals should consider including requests for gender parity and inclusion of local researchers prominently in manuscript authorship, while also including free of charge opportunities for mentorship and language support. New studies that measure the gender gap by area and the aggregation of data must be carried out to update knowledge and delve into other aspects, such as ethnicity, and go beyond the binary aspect of gender presented here. And finally, also to understand how and whether there are any implications to gender and affiliation of research leadership in studies relating to sexual and reproductive health and rights.

## Conclusions

Our article demonstrates that while there has been an increase in female authors in recent years, women are still behind men with regards to seniority and prestige. Likewise, female representation is closely tied to what institution they are affiliated with and where that institution is located. It is not acceptable to leave the change we want up to chance, the passing of time, or the luck of where women are located. Global health research institutions and especially other such special programmes for research need to actively promote change by ensuring women are included in research and research outputs, giving them opportunities to lead, and that local researchers are provided the leadership roles commensurate with their contributions. Institutions with a global reach must drive change and use privileged positions of power to create ways to encourage, finance, and disseminate mechanisms towards equality.

## Supporting information

S1 Table Strategy used for the database search. Conducted on 4 May 2022.
(DOCX)

S1 Fig Time series analysis of all authors excluding those affiliated with HRP in publications from 1972 to 2021, representing absolute and smoothed data, stratified by gender. (TIF)

S1 File Full dataset including all publications included in this analysis. (CSV)
