# Supplementary material for: Scientific production in sexual and reproductive health and rights research according to gender and affiliation: An analysis of publications from 1972 to 2021
Source: PLoS One. 2024 Jun 26;19(6):e0304659. doi: 10.1371/journal.pone.0304659 (PMC11207172; doi:10.1371/journal.pone.0304659)
Supplement: S1 Table — Conducted on 4 May 2022. (DOCX) [file pone.0304659.s001.docx]

**Scientific production in sexual and reproductive health and rights research according to gender and affiliation: a review from 1972 to 2021**

| **S1 Table. Strategy used for the database search (conducted on 4 May 2022)** |
| --- |
| *For PubMed*  ("Development and Research Training in Human Reproduction"[Affiliation]) OR ("Department of Reproductive Health and Research"[Affiliation]) OR ("Department of Sexual and Reproductive Health and Research"[Affiliation]) OR ("HRP"[Affiliation]) OR ("Human Reproduction Programme"[Affiliation]) OR (("Human Reproduction"[Affiliation] OR "Reproductive Health"[Affiliation] AND ("world health organization"[tw])) OR (("Human Reproduction"[tw] OR "Reproductive Health"[tw] AND ("world health organization"[tw])) |
| *For Web of Science*  ("Development and Research Training in Human Reproduction") OR ("Department of Reproductive Health and Research") OR ("Department of Sexual and Reproductive Health and Research") OR ("HRP") OR ("Human Reproduction Programme") (Affiliation) OR “Human Reproduction" OR "Reproductive Health" (Funding Agency) and “world health organization” (All fields) OR “Human Reproduction" OR "Reproductive Health" (All fields) and “world health organization” (Funding Agency) |
