# Supplementary figures and images for: Scientific production in sexual and reproductive health and rights research according to gender and affiliation: An analysis of publications from 1972 to 2021
Source: PLoS One. 2024 Jun 26;19(6):e0304659. doi: 10.1371/journal.pone.0304659 (PMC11207172; doi:10.1371/journal.pone.0304659)

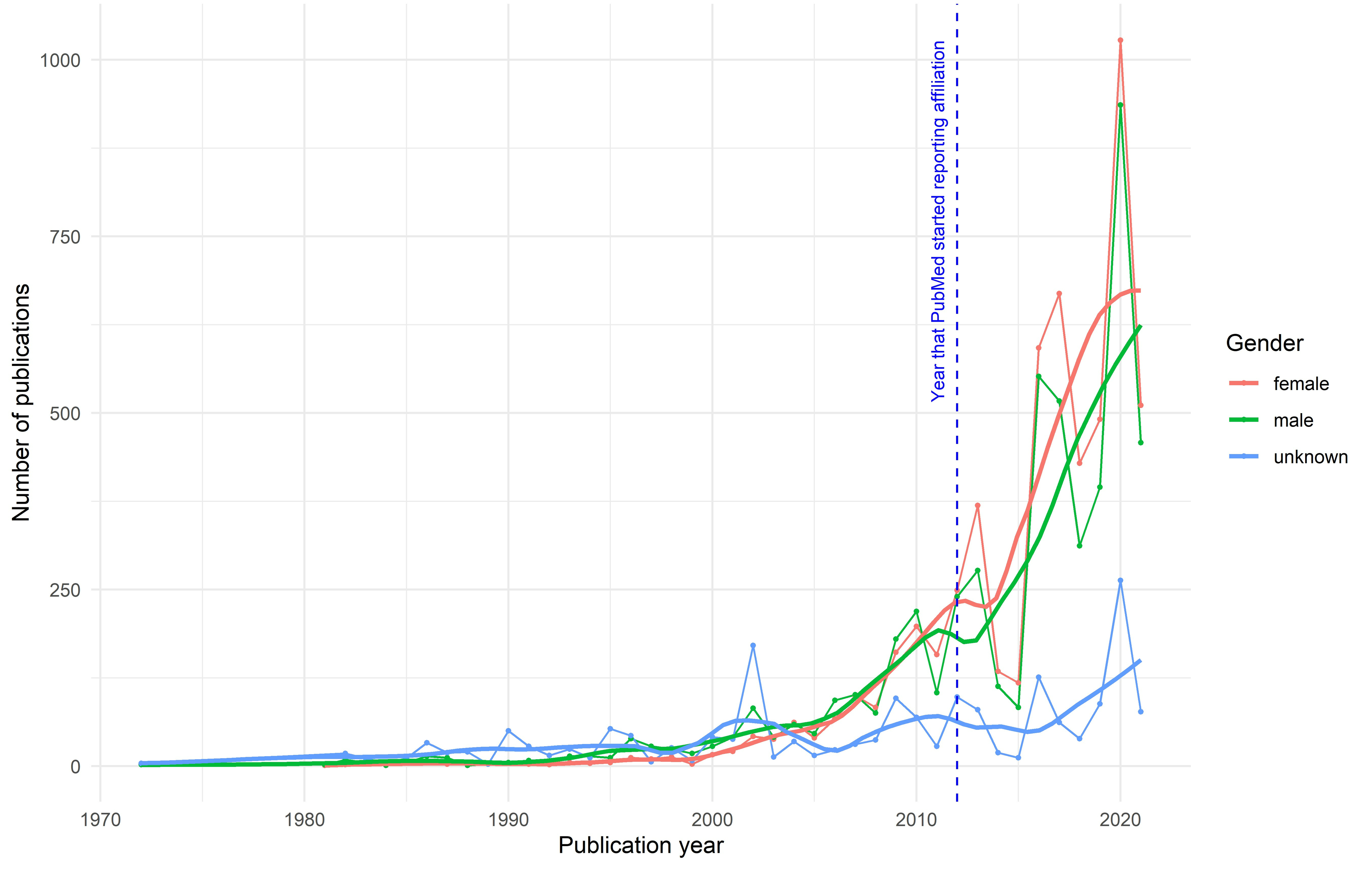

Supplement: S1 Fig — (TIF) [file pone.0304659.s002.tif]
